# Management on late diagnosed Fournier's gangrene in elderly patient and it's complication: A case reports

**DOI:** 10.1016/j.ijscr.2025.111888

**Published:** 2025-09-04

**Authors:** Nadya Rahmatika, Soetojo Wirjopranoto, Bagus Wibowo Soetojo, Yufi Aulia Azmi, Antonius Galih Pranesdha Putra, Kevin Muliawan Soetanto

**Affiliations:** a Departement of Pediatric, Faculty of Medicine, Universitas Airlangga, Surabaya, Indonesia; b Department of Urology, Faculty of Medicine Universitas Airlangga – Dr. Soetomo General Academic Hospital, Surabaya, Indonesia; c Departement of Orthopaedic and Traumatology, Faculty of Medicine, Universitas Airlangga – Dr. Soetomo General Academic Hospital, Indonesia; d Department of Health Sciences, University of Groningen, University Medical Center Groningen, Groningen, the Netherlands; e Department of Immunology, Faculty of Medicine Siriraj Hospital, Mahidol University, Bangkok, Thailand; f Department of Biomedical Science, Faculty of Medicine, Universitas Surabaya, Indonesia

**Keywords:** Fournier's gangrene, Mortality, Case report, Complication, Infection

## Abstract

**Introduction and importance:**

One of the risk factors linked to mortality in Fournier Gangrene (FG) is the elderly. When this risk is present and diagnosed too late, patient care may become difficult. This case report discusses the treatment of an older patient with late-diagnosed Fournier's gangrene and its consequences in this background.

**Case presentation:**

An emergency department (ER) referral was made for a 65-year-old male. For one week, the patient's main complaint was a sporadic high fever that got worse along with sporadic scrotal soreness. An X-ray of the kidney, ureter, and bladder (KUB) revealed gas accumulation and soft tissue oedema in the pelvic region. Fast-acting insulin was used to control blood sugar levels, and empirical antibiotic injections were used for initial care. A tunnel was discovered in the left inguinal area, and debridement was carried out right away, beginning with an incision in the necrotic area. Daily wound care was done routinely. Overall, the patient was doing well.

**Clinical discussion:**

This case highlights how crucial it is to diagnose Fournier's gangrene based on the radiological evaluation of a KUB X-ray. In this case, the KUB helped confirm gas gangrene because the patient had experienced acute scrotal swelling for 5 days, which was diagnosed late as FG. The initial physical examination revealed no crepitus or necrotic areas. FG is a clinical diagnosis, and imaging should not delay source control. Debridement necrotomy is the final step, and pharmacological and non-pharmacological measures must be taken promptly and concurrently. If type 2 diabetes mellitus is present, pharmacological measures include blood sugar control and the administration of double empirical antibiotics.

**Conclusion:**

An extensive physical examination, including investigations, is advised if an aged patient reports scrotal and testicular pain. Aggressive pharmaceutical and non-pharmacological treatment will be administered concurrently if the problem is discovered too late.

## Highlights

• Late-diagnosed Fournier's gangrene in elderly patients poses serious management challenges.
• Immediate empirical antibiotics and debridement-necrotomy needed in elderly patient.
• Early combined pharmacologic and surgical interventions improved patient outcomes.

## 1 Introduction

Fournier's gangrene (FG) is a necrotizing infection of the abdomen and perineum. FG is a rapidly spreading infection that penetrates the superficial and deep fascial layers in the perianal, genital, or perineal areas, leading to multiple organ failure and septic shock [1]. It was initially discovered in 1883 as necrotizing fasciitis of the perianal, perineal, and external genitalia in five of the patients treated by French dermatologist and venereal specialist Dr. Alfred Fournier [2]. The illness damages surrounding soft tissue by rapidly causing severe inflammatory and infectious processes to develop along fascial planes. Because early-stage Fournier gangrene has little or no cutaneous symptoms, it may go unnoticed or untreated [3].

Information on the prevalence and mortality of FG is essentially nonexistent. The southeastern United States has the highest documented rates of Fournier gangrene, with 1.9 cases per 100,000 inhabitants [4]. In these cases, hospital admissions of FG patients were rare [5]. As an uncommon condition, Fournier's gangrene is thought to occur in 1.6 out of every 100,000 males. A major observational study found that two-thirds of U.S. hospitals saw no cases on average per year, while just 6 % of hospitals treated three or more cases of Fournier's gangrene annually [4]. Despite its rarity, it is an extremely severe sickness with a high death rate, estimated to be between 4.7 and 40.4 %. [6].

A rapidly progressing, high-mortality condition, FG is frequently misdiagnosed due to the generic nature of its symptoms [1]. It is an actual medical and surgical emergency that requires multidisciplinary care [7]. Early risk factor identification and appropriate treatment are necessary to lower the fatality rates in high-risk FG patients [8]. Since it initiates an effective treatment, an early and accurate diagnosis affects the therapy's outcome [9].

Research has shown that an elderly person is one of the risk variables linked to death in FG [10]. Compared to men, people over 64, and people with other conditions, women had a slightly higher overall FG death rate [11,12]. When these risk factors are present and a diagnosis is delayed, patient treatment becomes difficult. It's crucial to keep in mind that effectively managing FG is difficult. This is due to the rapid progression of necrosis and the delayed diagnosis caused by confusing symptoms [1]. In one study, 8098 patients had necrotizing soft tissue infections of the genitalia. The findings showed that about 5096 (63 %) of the diagnostic visits with similar symptoms were caused by diagnostic delays [13].

Given this background, this case study details the treatment of elderly patients with late-diagnosed Fournier's gangrene and its complications, ultimately allowing the patient to live on. The SCARE Guidelines have been followed in reporting this case [14].

## 2 Case presentation

**Fig. 1:**
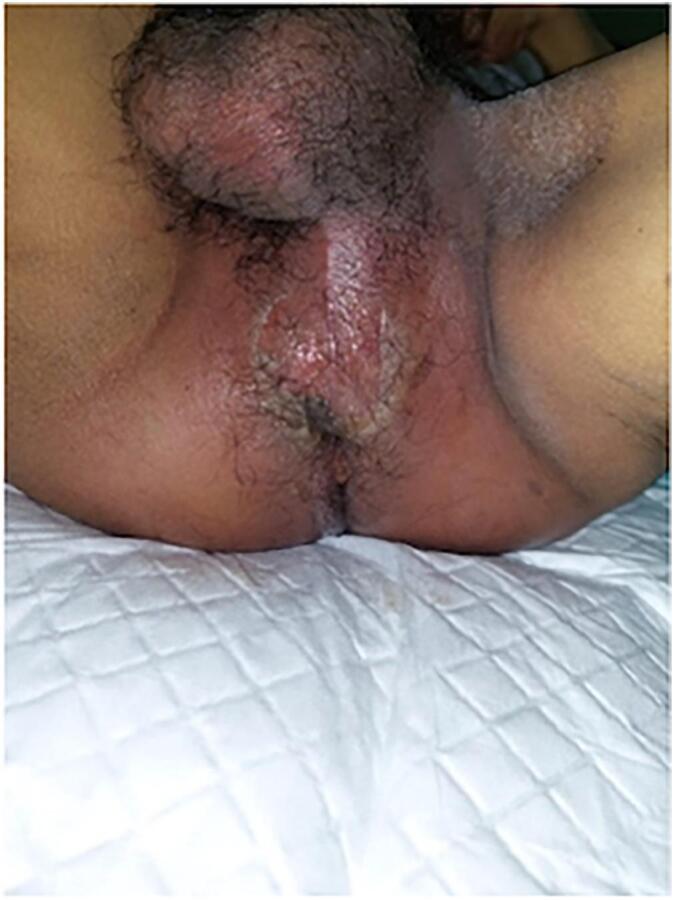
Clinical Picture of the Patient before hospital admission.

A 65-year-old man was referred to the emergency room (ER) of our hospital. The patient's main complaint was intermittent high fever accompanied by intermittent scrotal pain for 1 week before hospital admission. The fever and scrotal pain with a visual analog score (VAS 6). The scrotal pain has been felt intermittently since 5 days ago, it worsened 1 days before hospital admission (Fig. 1). The patient with a low education level only give antipyretics if the patient has had a fever and pain for the past 1 week.

**Fig. 2:**
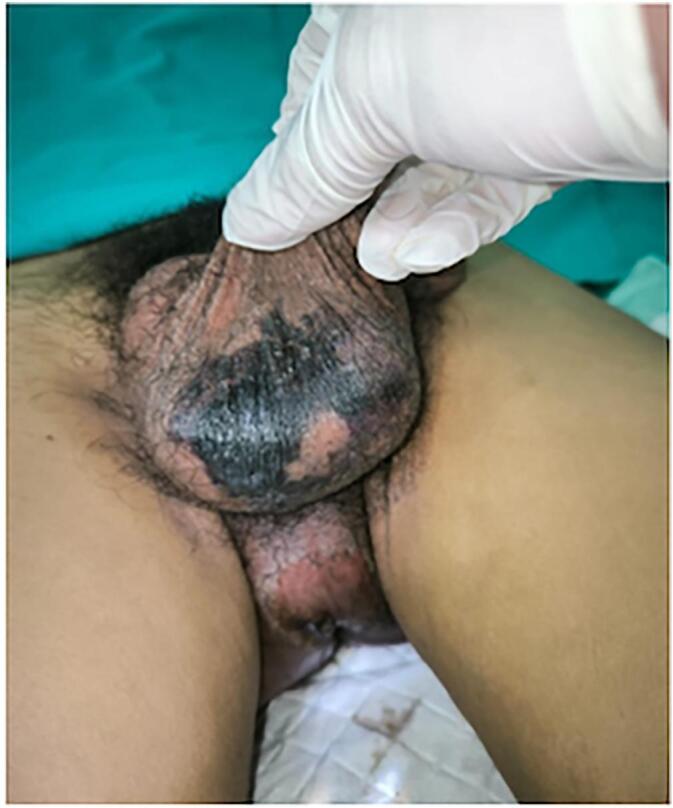
Clinical Picture of the Patient at the hospital.

**Fig. 3:**
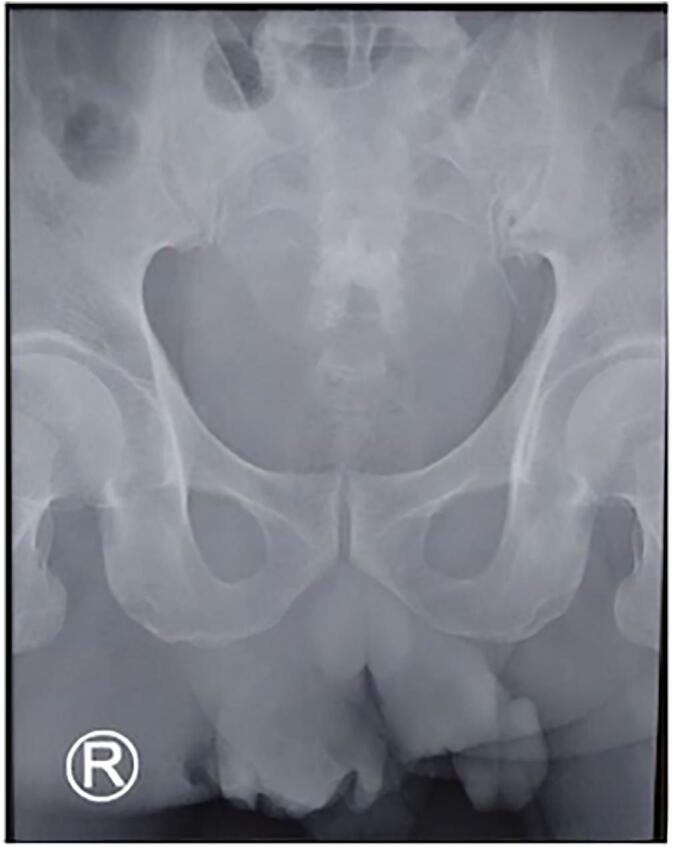
KUB X ray.

From the patient's physical examination in the ER, the body temperature was 41C, a mass at the perineum followed by a necrotic area (Fig. 2). The patient's condition was unstable, with a decrease in EVM (E: eye; V: verbal; M: movement) and blood pressure, contributing to the initial EVM of 456, which decreased to 233, and an initial blood pressure of 128/78 mmHg, which subsequently decreased to 75/50 mmHg. Ringer Lactate 2 L was inserted, Trendelenburg position, small incision in the scrotum, a double antibiotic was inserted, then necrotomy debridement was immediately performed for source control. The patient's laboratory results showed Haemoglobin (Hb) 12.1 g/dL, white blood cell (WBC) 32.59 × 10^3^ uL, creatinine serum 0.8 mg/dL, random blood sugar (RBS) 282 mg/dL, and CRP increased to 4.34. In this case report, the diagnosis of FG was confirmed by clinical examination, which revealed scrotal crepitations, and KUB = Kidney-Ureter-Bladder (plain radiograph) examination revealed gas gangrene. Under certain circumstances, a KUB radiological examination can be used to determine the presence of gas gangrene. The kidney, ureter, and bladder (KUB) x-ray examination showed soft tissue swelling and gas formation in the pelvic area (Fig. 3). The patient was diagnosed with a late diagnosis because he had been complaining of pain in his scrotum for the past 5 days. Clinically, he should have suspected FG and had supporting examinations such as an ultrasound performed, but this was not done; only painkillers were given. The FGSI score is calculated based on laboratory results and a physical examination (FGSI Score of this patient was 18).

**Fig. 4:**
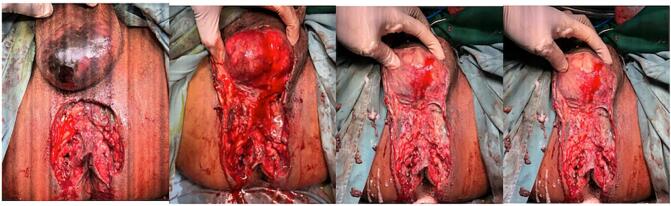
Clinical picture of debridement necrotomy procedure.

Initial management was performed with the injection of an empirical antibiotic, ceftriaxone 1 g every 12 h, and metronidazole 500 mg every 8 h intravenously (IV), and regulating blood sugar with rapid-acting insulin 3 times 12 international units (IU). Immediate debridement was performed, starting with an incision at the necrotic area. We also found tunnelling at the left inguinal (Fig. 4). The wound was washed with povidone iodine and NaCl 0.9 %, and wound care was performed routinely every day. For open wounds, regular daily open wound care is performed. After the wound is cleaned, silver sulfadiazine (SSD) and sorbacht are applied. No re-suturing is required. Tunnelling due to FG can be performed during surgery to explore for tunnelling to other areas.

**Fig. 5:**
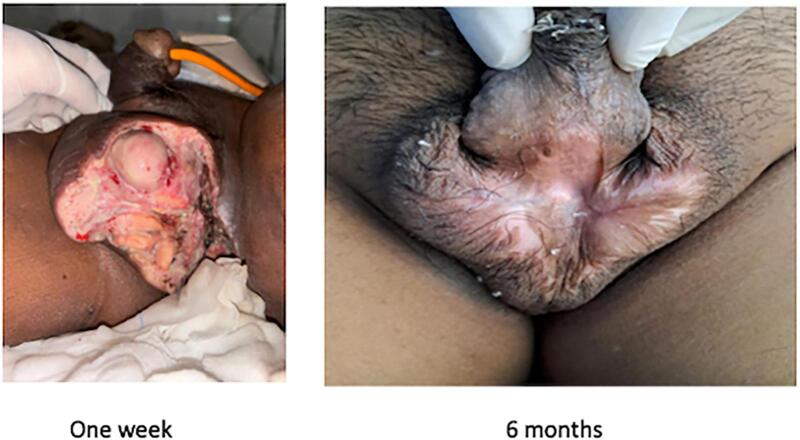
Clinical picture of wound healing.

The following day after surgery, the patient was in good general condition, with no complaints, good vital signs, and 700 cc/24-h urine production. He was discharged from the hospital on Day 2 post-operative. Weekly routine urology polyclinic visits for 1 month after hospital discharge showed good condition of the wound (Fig. 5).

The patient was discharged on post-op day 4. Before going home, a CBC test was performed, and the leukocyte count had dropped to 9.8 × 10^3^. The patient's family requested that he go home. In addition, the family had a nurse, and he underwent independent wound care at home. The catheter is kept in place for 2 weeks to prevent urine leakage.

## 3 Discussion

FG cases in the elderly increase mortality, especially in those with comorbid metabolic diseases such as diabetes mellitus, hypertension, and urinary tract infections. The patient in this case had a good prognosis. Another case report described a 65-year-old man with a history of self-care neglect, hypertension, and extensive tobacco use. The patient presented to the emergency department with classic symptoms of systemic disease, requiring a collaborative diagnostic and therapeutic approach. As a result of aggressive intervention, his clinical condition worsened throughout his 24-day hospitalization [15]. Other case series describe studies on older patients of different ages. A 69-year-old man with diabetes was diagnosed with Fournier's gangrene after he complained of excruciating scrotal and penile pain. In another case, a 91-year-old man who had previously experienced benign prostatic hyperplasia (BPH) developed gangrene quickly after presenting with ulcerative lesions and scrotal pain. The surgical strategy, which involved bilateral orchidectomy and several debridements, demonstrated the difficulty of treating Fournier's gangrene. The patient had improved, according to the results [16].

In this case, the patient improved with appropriate treatment despite a delay in diagnosis. Debridement necrotomy is the final step in this situation, particularly for diabetes mellitus type 2. The patient's healing process will be accelerated by proper postoperative wound care. Comprehensive wound debridement, antibiotic treatment, and appropriate diabetes management are the cornerstones of treating perianal abscess with Fournier's gangrene in diabetic patients [17]. Because diabetes mellitus impairs wound healing, chronic wounds are common in these people. Debridement is the term used to describe the surgical excision of diseased or dead tissue to stop the infection from spreading and to encourage recovery. Despite being a more general name, necrotomy also entails cutting into or extracting dead tissue and is a component of the larger debridement procedure in Fournier's gangrene [18]. This case explores the potential for preventing death in patients with late diagnosis of FG in the elderly, but this is only 1 case, which cannot be generalized in the population.

The mortality rates of patients with specific factors, such as acute renal failure, fewer operations (<3), respiratory failure requiring intubation, and polymicrobial wound infections, were higher than those of patients without these factors, according to another study evaluating the prognosis of 90 elderly patients with Fournier's gangrene. Early detection, vigorous resuscitation, the administration of broad-spectrum antibiotics, and timely and recurrent surgery are the cornerstones of managing Fournier's gangrene in elderly patients. By using rigorous treatment and a longer hospital stay, the medical staff can save their lives [19].

A prompt and accurate diagnosis, particularly through a physical examination followed by necrotizing debridement, can reduce the risk of mortality in elderly patients. In this case, the patient was diagnosed with a KUB X-Ray. Even though the diagnosis is mainly clinical, imaging can be used to confirm the diagnosis, assess the infection's severity, and pinpoint its origin. The characteristic of Fournier's gangrene is the formation of gas in the afflicted tissues as a result of bacterial infection. This gas is visible with KUB X-rays and shows up on the radiograph as dark, lucent areas [20]. *Clostridium perfringens* is the most prevalent, induces gas gangrene, a soft tissue infection that is extremely deadly [21]. KUB is the most popular method readily available, is less expensive, and exposes the patient to less radiation than CT [22]. KUB is widely used in urology cases and can be helpful in FG cases. Plain kidney-ureter-bladder (KUB) radiography is commonly used in cases such as urolithiasis, as most types of stones can be seen on KUB, although the sensitivity of KUB in detecting stones is limited (45–58 %). KUB has been reported to have a sensitivity of 96 % and a specificity of 91 % for diagnosing ureteral stones when combined with ultrasound. [23].

The best screening method is to use an MRI or CT scan, but the cost is high. In addition to additional time, expense, and radiation and contrast exposure, advanced imaging, such CT, is currently the gold standard for diagnosis, but it can be impractical because of the instability of many affected patients, as in the instance presented here [24]. The first assessment of the illness is called a radiological examination, which includes computed tomography (CT), magnetic resonance imaging (MRI), radiography, and ultrasound. However, FG is typically diagnosed clinically, and imaging might be useful when clinical symptoms are unclear or diagnoses are unclear [25]. Radiologic imaging may be useful if the diagnosis is ambiguous, but it shouldn't delay surgery. Even though imaging is typically a clinical diagnostic tool, it can be a helpful tool to aid in early diagnosis in uncertain cases. Because of its superior sensitivity, specificity, and soft tissue contrast, MRI remains the gold standard imaging method for NF assessment [26].

Pharmacological and non-pharmacological measures must be carried out immediately, simultaneously, pharmacologically with the administration of double empirical antibiotics and blood pressure regulation, if any. Early diagnosis, aggressive, thorough surgical treatment, and administration of the proper antibiotic treatment comprise the cornerstone for the outcome of this disease [27]. The prognosis is directly correlated with the time it takes to detect FG and the amount of time that passes before surgical debridement. Therefore, the care of FG should explicitly stress the significance of early surgical debridement supported by fecal diversion and skin repair when necessary, in addition to the combination of intensive fluid resuscitation and broad-spectrum antibiotics [28].

The plan for patients after catheter insertion is to undergo bladder training within two weeks before removal. Urethral catheterization, if not done following the standard steps, can cause urethral injury and be further complicated by necrotizing infection. In addition to the source control, suprapubic catheterization for urinary diversion is one of the management options if the source of the necrotizing fasciitis is a urologic source with urethral involvement [29]. In this patient, regular wound care is recommended, and after regular evaluations, the wound is improving. This patient-centered approach can help to reduce the need for burdensome wound care and increase the rate of patients returning home, allowing patients to recover sooner. To encourage tissue salvage and reduce the chance of disease progression, high-quality wound care is essential [30].

## 4 Conclusion

In cases of late diagnosed Fournier gangrene, pharmacological and non-pharmacological management must be carried out immediately to avoid patient mortality. If an elderly patient complains of scrotal and testicular pain, a thorough physical examination, including investigations, is recommended. If the condition is diagnosed late, aggressive pharmacological and non-pharmacological (surgical) treatment will be performed simultaneously.

## Ethical approval

Ethical approval for this study was provided by Health Research Ethics Committee of Dr. Soetomo General-Academic Hospital, Surabaya,

## Additional information

The information contained in this paper is personal.

## Declaration of competing interest

The author stated that there was no conflict of interest.

## Data Availability

The research outlined in the article does not make use of any data.
